# Conveying Movement in Music and Prosody

**DOI:** 10.1371/journal.pone.0076744

**Published:** 2013-10-16

**Authors:** Stephen C. Hedger, Howard C. Nusbaum, Berthold Hoeckner

**Affiliations:** 1 Department of Psychology, The University of Chicago, Chicago, Illinois, United States of America; 2 Department of Music, The University of Chicago, Chicago, Illinois, United States of America; University of California, San Francisco, United States of America

## Abstract

We investigated whether acoustic variation of musical properties can analogically convey descriptive information about an object. Specifically, we tested whether information from the temporal structure in music interacts with perception of a visual image to form an analog perceptual representation as a natural part of music perception. In Experiment 1, listeners heard music with an accelerating or decelerating temporal pattern, and then saw a picture of a still or moving object and decided whether it was animate or inanimate – a task unrelated to the patterning of the music. Object classification was faster when musical motion matched visually depicted motion. In Experiment 2, participants heard spoken sentences that were accompanied by accelerating or decelerating music, and then were presented with a picture of a still or moving object. When motion information in the music matched motion information in the picture, participants were similarly faster to respond. Fast and slow temporal patterns without acceleration and deceleration, however, did not make participants faster when they saw a picture depicting congruent motion information (Experiment 3), suggesting that understanding temporal structure information in music may depend on specific metaphors about motion in music. Taken together, these results suggest that visuo-spatial referential information can be analogically conveyed and represented by music and can be integrated with speech or influence the understanding of speech.

## Introduction

Throughout the history of Western music, composers, who conceive of music as a way to communicate meaning, have intentionally exploited the structural similarities between the acoustic properties of music and the patterns of events in the world cf. [1]. Generally referred to as extramusical meaning [2], [3], these composers have written passages that assumed a metaphoric relationship between some acoustic properties of music and properties of non-musical events or objects. For example, musical tempo changes have often been used to suggest physical motion. In Renaissance madrigals, the idea of running is musically depicted in the use of quickly occurring groups of notes against a slower context; in Arthur Honegger’s orchestral work *Pacific 231*, the movement of a steam engine is portrayed through a gradual acceleration of tempo. Rimsky-Korsakov’s *Flight of the Bumblebee*, in which sixteenth notes are played almost continuously at over 140 beats-per-minute, often accompanies rapid visual motion in multimedia contexts.

This association between metaphoric motion depictions in music and physical motion has recently been empirically investigated. Eitan and Granot [4] asked participants to imagine how a human character would move to simple musical motifs, including accelerating and decelerating motifs. The authors showed that accelerating and decelerating motifs elicited images of increasingly and decreasingly speeded motion, respectively. Their study implies that certain musical properties, even in the absence of visual information, are interpreted metaphorically, that is, they reliably activate mental images of visual motion. The relationship between acceleration and deceleration in music and mental images of speed were also found in children [5], although the association was not as strong, suggesting a potential learned influence to the metaphoric mapping.

Music is not the only instance in which auditory pattern properties may convey meaning. Although linguists generally hold that patterns of speech are arbitrary with respect to meaning, these patterns may serve as a ground for establishing meaning correspondence between words even across languages [6]. Recent research has also shown that temporal patterns can convey descriptive information about object movement. Shintel, Nusbaum, and Okrent [7] demonstrated that in describing the direction of a moving dot on a screen using words, participants changed their speaking rate to illustrate the relative speed of dot motion, while a different group of participants could use the temporal cues of the spoken sentences to infer the dot speed.

The ability to quickly map acoustic patterns onto other dimensions in categorization tasks might be explained in terms of perceptual representations or symbols [8]. Perceptual symbols can be thought of as the residues of perceptual experiences, which are stored as patterns of activation in the brain [9]. Instead of assuming that mental representations are amodal and abstract, the idea of perceptual representations assumes that our sensorimotor experiences with particular objects in different contexts will influence how we remember and use even abstract category information, in a process called simulation [10]. For example, in imagining a dog, one would re-experience the previous sensory attributes of experiences with dogs. Thus, someone who has watched a lot of greyhound racing would have a much different mental representation than someone who has trained seeing-eye dogs with respect to the shape and movement of dogs.

Perceptual symbols have also been used to explain aspects of sentence understanding. For example, expert hockey players activate motor cortex during comprehension of hockey action sentences, which does not occur for non-hockey players [11]. Zwaan et al. [9] demonstrated that participants were faster to respond to pictures of objects mentioned in sentences when the shape of the object was implied by the sentence, albeit irrelevant to the task. For example, in the sentence, “The ranger saw the eagle in the sky”, participants were faster to respond to a picture of an eagle with its wings outstretched (compared to a picture of an eagle with its wings folded). In understanding the sentence, participants are presumably faster to recognize the eagle with outstretched wings compared to folded wings because the former is consistent with the perceptual representation or image created as part of understanding the sentence about a flying eagle. Similarly, Shintel and Nusbaum [12] showed that the speaking rate of a sentence influenced the response speed for recognition of a subsequently viewed picture, in that fast speaking rates facilitated the comprehension of pictures implying motion (e.g., a picture of a galloping horse), while slow speaking rates facilitated the comprehension of pictures implying rest (e.g., a horse standing at a stable). As in the Zwaan et al. [9] study, these results suggest that hearing fast or slow speech conveyed something about the movement of the to-be-recognized horse even though motion was never mentioned. This suggests that listeners create an image or perceptual representation of a horse in motion when hearing fast speech or a horse at rest when hearing slow speech. These studies on perceptual pattern processing make use of structural similarities across modalities similar to the conceptual work of metaphor in language, which is now seen as a central process in understanding rather than a supplementary inferential process [13]. This suggests the possibility that a descriptive mapping using a metaphoric relationship may serve a direct communicative role in both speech and music. In the present study, therefore, we investigated whether listeners use the metaphoric motion information in music as a natural part of perception - something that has been previously found in speech [12]. Specifically, we address whether temporal structure in music interacts with the perception of a visual image to spontaneously form an analog perceptual representation. We hypothesize that listening to varying the speed of music, in the context of a categorization task, will result in participants’ imagining motion, which in turn will facilitate responses to objects that are pictured in motion. Similarly, we hypothesize that listening to decelerating music will result in simulations of an object slowing down or stopping, and thus participants should be faster at responding to objects that are pictured at rest.

## Experiment 1

The first experiment addressed whether listeners implicitly interpret temporal patterns in music as motion information, even when neither the music nor the concept of movement is an integral or explicit part of the task. The idea that listeners associate acoustic properties with changes in other sensory domains – particularly vision – has been well researched [14], [15]. These audiovisual studies, however, rely on conscious and explicit decisions about variation in one modality (e.g., loudness), with the other modality providing congruent – and task-irrelevant – information (e.g., size). In other words, consciously attending to the relevant dimension in one modality was necessary to perform the task.

The present studies, in contrast, explore whether the associations between motion information in audition and vision influence performance on a task in which *neither* cue is necessary for successful performance. Thus, we designed a task in which the criterion for responding to a stimulus was independent of the motion information in the music: participants were asked to categorize pictures of objects (e.g., dog, person, baseball) as either animate or inanimate. In all pictures animacy and motion were independent so that motion was orthogonal to the goal of this task: a baseball in flight is no more animate than one at rest and a standing dog is no more inanimate than a running dog. Moreover, participants could completely ignore the musical information on each trial and perform the experimental task with 100% accuracy since the music did not cue the judgment in any way. The main question was thus: even though the motion information contained within the music and the pictures was unrelated to the task, would listeners use this information in their judgments of animacy? If the metaphoric motion represented in the music influences the perceptual understanding of visually depicted objects, we hypothesized that participants would be faster at responding when the motion information in the music is congruent with, and thus anticipating, motion information represented within the picture.

### Methods

#### Participants

Twenty University of Chicago students [12 female, mean 20.4± SD 1.6 years, age range 18–24] participated in the experiment. All participants had no known hearing loss and normal or corrected-to-normal vision. This research was approved by the University of Chicago’s IRB. All participants gave written consent prior to participating in the experiment. Participants were compensated for their participation in the experiment.

#### Materials

The test items included images of 10 animate objects (e.g., cat, horse, dog) and 10 inanimate objects (e.g., train, car, baseball). Each item was represented both in motion and at rest, resulting in 40 total images. The music motif stimuli were created with a MIDI controller, using Reason 4.0 synthesis. These motifs consisted of two oscillating notes (C4 and D4) - a musical gesture commonly referred to as a trill and often occurring alone in musical pieces. The trills were either accelerating or decelerating. The accelerating motifs began with each note being played 500 ms apart (or a tempo of 120 BPM) and ended with each note being played 100 ms apart (or a tempo of 600 BPM). The decelerating motifs began at 600 BPM and ended at 120 BPM. Participants listened to the music motifs using Sennheiser HD-280 headphones. The experiment was controlled on a computer using E-Prime 2.0.

#### Procedure

**Figure 1:**
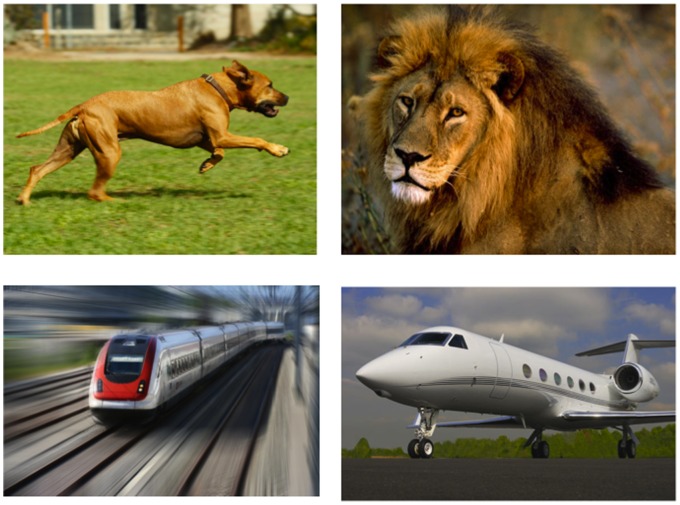
Sample pictures from Experiment 1, in which participants made animacy judgments independent of motion cues. *Top left*: animate, motion. *Top right*: animate, rest. *Bottom left*: inanimate, motion. *Bottom right*: inanimate, rest.

On each trial, participants first heard the accelerating or decelerating music motif, and then were presented with a picture. Participants were instructed to decide whether a picture that appeared on the computer screen was animate or inanimate as quickly and as accurately as possible. If the object was animate, participants pressed a button labeled “A” on the computer keyboard. If the object was inanimate, participants pressed a button labeled “I”, located on the opposite end of the keyboard. The location of the response buttons was counterbalanced between participants. Each image either depicted an animate or inanimate object in motion or at rest. The images were completely crossed, in that each participant saw an equal number of animate and inanimate objects in motion and at rest. Even though both “motion” and “rest” images were static, in that no real motion was used, previous studies have demonstrated that static images of objects can imply motion [16]–[18]. Figure 1 provides an example of the visual stimuli that were used during the experiment. Since motion was never mentioned with regard to the music or the pictures, participants were led to believe that the experiment was solely about categorization.

After the experiment ended, participants were debriefed and compensated for their participation. During debriefing, we asked participants to guess the purpose of the experiment. While some participants mentioned motion in their response (e.g. thinking that “fast” music would make one respond faster overall), no one correctly identified the purpose of the experiment (thinking that the motion congruence between the music and picture would make one respond faster overall).

### Results

#### Linear Mixed-Effects Model

To find out whether participants were faster at responding when the motion information in the music was congruent with motion information represented within the picture, we compared the mean response times of the two congruent conditions (accelerating music/object in motion and decelerating music/object at rest) to the response times of the two incongruent conditions (accelerating music/object at rest and decelerating music/object in motion). Indeed, mean response times for the congruent trials (M = 589 ms, SEM = 33 ms for accelerating music/object in motion and M = 572 ms, SEM = 30 ms for decelerating music/object at rest) were faster than response times for the incongruent trials (M = 608 ms, SEM = 36 ms for accelerating music/object at rest, and M = 614 ms, SEM = 38 ms for decelerating music/object in motion).

To test whether these mean differences were statistically different, we followed the guidelines set forth by Baayen, Davidson, and Bates [19] for analyzing data with both fixed and random effects. Specifically, we ran a mixed linear model with musical motion (accelerating versus decelerating), object motion (implied motion or rest), and image animacy (animate versus inanimate) as fixed effects. Images and participants were treated as crossed random effects, and we used the maximal random effects structure justified by the design [20]. We found a significant interaction between musical motion and object motion [Decelerating Music/Object Rest: *t* = −2.51, *p = *0.02]. Based on the means for the congruent and incongruent motion conditions, we were able to conclude that participants were faster to respond only when the motion information in the music matched the motion information in the picture.

Importantly, there were no main effects of musical motion [Decelerating Music: *t = *1.46, *p = *0.16] or object motion [Object Rest: *t = *1.36, *p = *0.19]. This means that musical motion and image motion did not independently affect response times. For example, participants did not become faster overall when hearing an accelerating music motif or viewing a picture that implied motion, as suggested in some (10%) of the participants’ inferences about the purpose of the study. Rather, it was only when the motion information in the music *matched* the motion information in the picture that participants became faster. Additionally, there was no main effect of animacy on response times [Inanimacy: *t = *1.34, *p = *0.20], suggesting that participants were not overall faster or slower to respond based on whether the object to be categorized was animate or inanimate.

Given that the task was an animacy judgment, we analyzed the data to find out whether participants had used accelerating music as a signal for animacy and decelerating music as a signal for inanimacy. We thus looked for the possible congruence of music to animacy, in which accelerating music might speed responses to animate objects, and decelerating music might speed responses to inanimate objects (rather than object motion and rest, respectively). This congruence of music and animacy, however, was not the case [Decelerating Music/Inanimacy: *t* = −0.84, *p = *0.41]. Thus, even though the task was specifically focused on animacy determinations for pictures and the metaphoric mapping of acceleration/deceleration to animacy could possibly have been made, participants’ responses reflected a predominant influence of the more standard cultural musical metaphor of temporal structure to motion.

#### Repeated-measures ANOVA

In addition to running a linear mixed effects model, we also used a repeated-measures analysis of variance with animacy (animate versus inanimate), music motion (accelerating versus decelerating), and object motion (implied motion versus implied rest) as repeated factors. The reason we include this separate test for significance is to see the degree to which both statistical analyses converge. Similar to the mixed effects model, we found a significant interaction between music motion and object motion [*F* (1, 19) = 8.89, *p* = 0.008, η^2^_p_ = 0.31], but no main effects of music motion [*F* (1, 19) = 1.16, *p* = 0.29, η^2^_p_ = 0.06], object motion [*F* (1, 19) = 0.47, *p* = 0.49, η^2^_p_ = 0.02], animacy [*F* (1, 19) = 1.81, *p* = 0.20, η^2^_p_ = 0.09], or a significant interaction between animacy and music motion [*F* (1, 19) = 0.69, *p* = 0.42, η^2^_p_ = 0.04].

### Discussion

Experiment 1 tested whether listeners use the metaphoric motion information conveyed by accelerating and decelerating music in understanding the visual representation of an object. The results showed that participants were faster to respond when the metaphoric motion in the music matched the motion in the picture.

The task was explicitly defined as an animacy judgment and the interaction of music type with depicted motion in the images was not part of the task at all. Since motion was irrelevant to the task, and since no participant correctly identified the purpose of the experiment, the interaction of music type with depicted motion may be due to the process of interpreting the image in the context of the music. Specifically, participants could have used visual mental imagery of motion [4] when listening to the music motifs to anticipate and understand the visual image following the music, thereby influencing recognition when the object in the picture was moving at a congruent speed with participants’ mental images. A related possibility is that through consistent cross-modal associations, which are learned culturally (e.g. through television, films, text painting in songs, etc.), participants have automatized the mapping between auditory and visual motion. Both interpretations would support the existence of shared cognitive and perceptual mechanisms for the processing of metaphoric auditory motion and visual motion.

## Experiment 2

While Experiment 1 provides evidence that acceleration cues in music can affect interpretation of motion in a static image, this does not necessarily mean that the processing of musical motion relies on similar mechanisms as the processing of analog acoustic information in other auditory realms, such as speech [7]. In speech, analog variation in prosody is conveyed concomitantly as spoken words. Compared to this literal integration of analog acoustic information with words in sentences, the perception of a sentence accompanied by music presents two discrete and separate sound sources (music does not normally accompany speech) and thus potentially presents a situation that could require divided attention. However, previous studies have shown that listeners are able to divide attention relatively easily and process some information in speech and nonspeech signals simultaneously [21]. It is important to note that in those studies, listeners were not doing much more than detecting a nonspeech signal presence and were not required to carry out pattern interpretation of both signals simultaneously. Since speaking rate and tempo are inherent parts of speech and music, respectively, it might be the case that motion signals represented in one domain can influence the formation of a perceptual representation, even if attention to that domain is not necessary for the task or even explicitly related in any way to the task. On the other hand, by simultaneously presenting two signals – one carrying metaphoric analogical motion information (music), and the other carrying lexical-semantic information (speech) which is *necessary* to attend to in order to successfully perform the task – it might be the case that the motion information in the music will not influence the formation of a perceptual representation or interpretation of the sentence because the music is streamed off as irrelevant to comprehension, unlike prosody which would be integral to the speech. Through pairing motion information in music with lexical-semantic information in speech, we tested these possibilities in the second experiment.

Similar to Experiment 1, we used a paradigm in which participants made judgments that were independent of the motion information contained within the music and the picture, but in this study the task judgments depended on the relationship between the linguistic message of a sentence and the content of a subsequent picture (a message that varied from trial to trial as a description of the object in the picture, unlike the single classification judgment in Experiment 1). Participants made decisions about visual object attributes such as color (e.g., responding to a picture of a car following a sentence such as “The car is red”), and thus participants could ignore any motion information in the accompanying music as well as in the picture and still perform the categorization task with 100% accuracy [9], using the same linguistic materials, instructions, and design as Shintel and Nusbaum [12]. If the irrelevant metaphorical motion information implied by the temporal structure of the music influenced participants’ perceptual processing of visual objects, then we would expect faster response times on trials in which the motion information in the music was congruent with the motion information in the picture.

### Methods

#### Participants

Twenty-eight University of Chicago students [15 female, mean: 20.9±SD: 2.4 years, age range 18–28] participated in the study. Two participants were not analyzed as they reported a history of hearing impairment, leaving twenty-six in the final analysis. All other participants reported no history of speech or hearing disorders, and had normal or corrected-to-normal vision. This research was approved by the University of Chicago’s IRB. All participants gave written consent prior to participating in the experiment. Participants were compensated for their participation in the experiment.

#### Materials

Test stimuli included 16 sentences that described different objects. None of the sentences used any words to describe motion and none implied that the described object was moving or not moving. Either of two pictures, which depicted the object described by the sentence, could be paired with each sentence, although both pictures were never displayed to the same participant. One of the pictures showed the object in motion and the other picture showed the object at rest. Additionally, 16 filler sentences were paired with 16 pictures that did not depict the object described in the sentence. The sentences, which were previously recorded and used by Shintel & Nusbaum [12], were spoken at a neutral speaking rate (mean words-per-minute rate of 193). The music stimuli were identical to those used in Experiment 1. Speech and music files were normalized and combined into a single waveform, in which the music accompanied the speech.

#### Procedure

**Figure 2:**
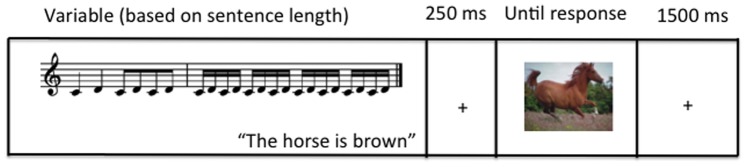
Sample trial from Experiment 2. In this particular trial, the musical motion (acceleration) matches the implied motion of the picture.

This experiment used the same linguistic materials, instructions, and design as were used in the study reported by Shintel and Nusbaum [12]. Participants were instructed to decide whether a picture represented an object that had been mentioned in the immediately preceding sentence, in a variant of a procedure first used by Zwaan et al. [9]. Participants only had to make a match judgment, and there was no required or even mentioned use of motion information. Each sentence was accompanied by either an accelerating or decelerating music motif, and was followed by a picture of the described object or a different object. For example, a sentence such as, “The horse is brown” could either be followed by a picture of a brown horse or another object. For the trials in which the object in the picture matched the object mentioned in the sentence, some participants saw the object depicted in motion, while other participants saw the object at rest. Figure 2 graphically depicts a sample trial from the experiment.

After the auditory stimuli (sentence accompanied by music) ended, participants saw a fixation cross appear on the screen for 250 ms, after which an image appeared in the center of the screen. Participants then had to determine whether the image was mentioned in the preceding sentence by pressing keys on a keyboard labeled “Yes” and “No”. The “Yes” and “No” keys were always on opposite ends of the keyboard and were counterbalanced. Participants were instructed to respond “Yes” if the image belonged to the same category as the described object in the sentence (e.g. if the sentence mentioned a car and the picture displayed a car); participants were led to believe that this was a categorization task as no sentences mentioned any motion cues.

### Results

#### Linear Mixed-Effects Model (LMEM)

For trials in which musical motion was congruent with object motion, participants were faster (mean ± SEM: 563 ms±38 ms for accelerating music/object in motion, and 549 ms±29 ms for decelerating music/object at rest) at responding compared to trials in which musical motion was incongruent with object motion (mean ± SEM: 611 ms±33 ms for accelerating music/object at rest, and 637 ms±54 ms for decelerating music/object in motion).

We tested the difference between condition means using a mixed-effects linear model with musical motion and object motion as fixed, repeated factors. Items and participants were treated as fully specified and crossed random effects. We found a significant interaction between musical motion and object motion [Decelerating Music/Object Rest: *t* = −2.18, *p*<0.05], suggesting that participants were able to use the musical motion information in conjunction with the lexical information in the sentence to form a perceptual representation of an object.

Similar to Experiment 1, there was no significant main effect of music speed [Decelerating Music: *t = *1.70, *p = *.10] or image motion [Object Rest:, *t = *1.07, *p = *0.30], meaning that participants did not simply show a priming effect of speed (i.e. responding fastest when listening to accelerating music irrespective of the implied picture motion or responding fastest when responding to pictures in motion irrespective of the music speed). Again, it was only when the motion information in the music *matched* the motion information in the picture (accelerating/object in motion and decelerating/object at rest) that participants responded fastest.

#### Repeated-measures ANOVA

We found converging results using an analysis of variance with music motion (accelerating versus decelerating) and object motion (implied motion versus implied rest) as repeated factors. Specifically, we observed a significant interaction between music motion and object motion [*F* (1, 24) = 13.96, *p*<0.01, η^2^_p_ = 0.32], but no significant main effects of music motion [*F* (1, 24) = 0.07, *p* = 0.80, η^2^_p_<0.01] or object motion [*F* (1, 24) = 0.38, *p* = 0.54, η^2^_p_ = 0.02].

### Discussion

Experiment 2 tested whether listeners could use metaphoric motion information represented by music while listening to spoken sentences. Unlike Experiment 1, in which participants did not need to attend to any auditory information to perform the animacy judgment task, in Experiment 2 the task required participants to actively listen to each sentence to understand the object description for comparison to the object in the picture. There was therefore reason to believe that participants might selectively attend to the speech signal while actively filtering out the music [22]–[24]. The fact that participants were able to perform the categorization task quickly with a high degree of accuracy while also showing the effect of the music suggests that listeners can extract with relative ease information from simultaneously presented speech and non-speech signals. It still remains unclear, however, whether listeners were integrating the motion information (e.g., acceleration) from the music with the lexical-semantic information (e.g., car) from the speech in order to form a unified perceptual representation (e.g., fast car), since our results could have also been obtained if the music and speech signals were processed separately and responses were simply influenced by both. Future research should address this issue, as it is theoretically important for understanding how cross-modal perceptual representations are formed. In any case, it is clear that in spite of the separation of underscoring music from the speech, in comparison to prosody, the music can still influence interpretation of the sentence.

While acceleration and deceleration have been extensively used in Western music as cues for object motion because they allow listeners to experience the tempo change dynamically, static tempo differences are also extensively used to convey object motion in generating extramusical meaning. Indeed, Shintel and Nusbaum [12] used fast and slow speaking rates, not acceleration or deceleration, to reliably convey motion information in a speech signal, so that this difference of the overall speaking rate is more akin to simple tempo differences in music. If the results from our two experiments so far can be explained through low-level perceptual priming mechanisms, we wondered whether statically fast or slow music motifs would have the same effect as accelerating and decelerating motifs. On the other hand, if the change in speed in the two previous experiments was based on an interpretation of the cultural meaning of musical information, then statically fast and slow music accompaniment to an unchanging speech rate might be different from fast and slow speech. Thus, in the third and final experiment we tested whether musical information was integrated into a perceptual representation when static tempo difference, rather than dynamic tempo changes, was used as a motion cue.

## Experiment 3

If the same temporal patterns of music and speech convey the same information about motion – and thus are integrated into perceptual representation – we hypothesized that manipulating the overall static tempo for music similar to the different speech rates used by Shintel and Nusbaum [12] should produce speed-motion congruence effects similar to the results of Experiment 2.

We tested this in a third experiment using the same sentences, pictures, and design used in Experiment 2 only changing the musical accompaniment to convey statically different tempos.

### Method

#### Participants

Twenty-one University of Chicago undergraduates [14 female, mean ± SD: 20.6±2.1 years, age range 18–25] participated in the study. All participants reported no history of speech or hearing disorders. This research was approved by the University of Chicago’s IRB. All participants gave written consent prior to participating in the experiment. Participants received course credit for their participation.

#### Materials

The materials were identical to Experiment 2 with the exception of the music stimuli, which were comprised of a single musical motif (quarter note oscillation between C4 and D4) played at either a fast (600 BPM) or slow (120 BPM) tempo. These tempos were matched in rate to the accelerating and decelerating trills of Experiments 1 and 2, which had end points of 120 BPM and 600 BPM. The music was generated, normalized, and combined with the spoken sentences in an identical manner to Experiment 2.

#### Procedure

The procedure was identical to Experiment 2. Participants heard sentences describing objects, which were simultaneously accompanied by statically fast or slow music motifs. After the sentence/music combination ended, participants were presented with an image of an object, which was depicted either in motion or at rest.

### Results

#### Linear Mixed-Effects Model (LMEM)

Similar to Experiments 1 and 2, we expected faster response times on trials in which the motion information in the music was congruent with the motion information in the picture; however, unlike Experiments 1 and 2, we did not find this result (see Table 1 for means and SEMs). To statistically test the condition mean differences (fast music/picture motion, fast music/picture rest, slow music/picture motion, slow music/picture rest), we used a mixed-effects linear model with musical motion and object motion as fixed, repeated factors. Items and participants were treated as crossed, random effects, and were maximally specified based on the experimental paradigm. The interaction between musical motion and object motion was not statistically significant [Slow Music/Object Rest: *t* = 0.90, *p = *0.38]. Thus, we did not find any evidence that participants used tempo cues as a marker of motion in their construction of a perceptual representation. Additionally, we did not find any main effects of music speed [Slow Music: *t* = −0.52, *p = *0.61] or object motion [Object Rest: *t* = −0.94, *p = *0.36].

**Table 1:** Mean response time (in milliseconds) per condition for the three experiments.

	*Music Motion*		*Music Rest*	
	PictureMotion	Picture Rest	PictureMotion	Picture Rest
Exp.1	563	611	637	549
	(38)	(33)	(54)	(29)
Exp.2	559	608	629	550
	(22)	(31)	(28)	(18)
Exp.3	607	608	572	626
	(27)	(37)	(34)	(39)

Experiments 1 and 2 exhibit significantly faster response times when the implied musical motion matches the implied picture motion (columns 1 and 4), while Experiment 3 does not. Standard error measurements are represented in parentheses.

#### Repeated-measures ANOVA

The results from an analysis of variance with music motion (fast tempo versus slow tempo) and object motion (implied motion versus implied rest) converged with the LMEM analyses, in that we failed to find a significant interaction between music motion and object motion [*F* (1, 20) = 2.31, *p* = 0.14, η^2^_p_ = 0.10]. We also did not find evidence for a main effect of music motion [*F* (1, 20) = 0.27, *p* = 0.61, η^2^_p_ = 0.01] or object motion [*F* (1, 20) = 1.49, *p* = 0.24, η^2^_p_ = 0.01].

### Discussion

The failure to find a speed effect using the same materials as Shintel and Nusbaum [12] suggests that music may not necessarily convey the same kind of motion information as prosody in speech. In speech, fast and slow speaking rates – which are analogous to the static tempo changes used in Experiment 3– have been reliably shown to convey object motion. Shintel et al. [7] first demonstrated that speakers vary speaking rate to describe speed of motion, and that listeners use this speaking rate to infer object speed. Shintel and Nusbaum [12] demonstrated that rate information in speech affects perception of static visual depictions of motion even when the task has nothing to do with motion perception (as in Experiment 2 with music). Shintel and Nusbaum [25] subsequently demonstrated that speaking rate information of an utterance is interpreted as motion information that can be qualified by the context of the meaning of an antecedent narrative. These three separate studies using very different methods have all found that speaking rate conveys information to a listener about motion. The results of Experiment 3, however, suggest that the way motion information is conveyed in analog expression in speech and in music is different. While analog variation of acoustic properties in speech and music can have the same effect on forming a perceptual representation, this effect is achieved through different metaphoric mappings of temporal pattern onto the concept of motion and rest.

One reason why statically fast and slow music motifs behave differently than dynamically accelerating and decelerating music motifs may be the contextual nature of the representation of speed. In the first case, an auditory event might need to be juxtaposed with a second auditory event in order to be accurately labeled as “fast” or “slow”. Here a comparison *between* stimuli is necessary to determine speed. In the second case, this juxtaposition and comparison is inherently built into accelerating and decelerating motifs, as notes become either more or less densely spaced *within* a single auditory stimulus. Previous research on speed adaptation has shown that the perceived speed of a constantly moving object is diminished, and moreover this reduction in perceived speed declines in an exponential manner as a function of stimulus duration [26]. While these studies have examined the relativity of speed in vision, it may be possible that music – which invokes motion information in a metaphoric way – adheres to the same perceptual mechanisms.

## Conclusion

The purpose of the current studies was to investigate the relationship between temporal patterns in music and the concept of object motion, and to situate this relationship in a broader communicative framework than has been done in previous research. Specifically, we wanted to know whether the temporal properties of music could – like speech – convey motion information about an object. In the first two experiments, we found that the metaphoric motion represented in music influenced participants’ interpretation of the visual representations of objects, providing evidence that temporal cues which are clearly separate from speech can nevertheless convey motion information about an object simultaneously described in speech. Experiment 3, however, suggests that this musical representation of motion does not seem to be solely driven by lower-level perceptual processes: unlike dynamically accelerating and decelerating motifs, statically fast and slow motifs did not influence visual judgments of object motion in a congruent fashion.

The significant difference in response times between congruent and incongruent trials in Experiments 1 and 2 demonstrates that participants’ judgments about objects were influenced by music even though the music was not explicitly part of the task nor was the motion of the visual objects directly relevant. In the second experiment, where the music was not integrated into the acoustic waveform of the speech and thus was clearly heard as a separate signal source, the effect of musical accompaniment was nevertheless consistent with the effects of speech rate on speech understanding reported by Shintel and Nusbaum [12] as if the music was conveying a message about the motion of the object described in the sentence. Moreover, this extraction of motion information in music appears to be a natural part of perception, as there were no instructions to attend to the musical information and in fact it had no relevance to the task at all. Indeed, a majority of participants (57% of participants across all three experiments) even found the music “distracting” or “annoying” upon debriefing, reporting that they tried to ignore it. Participants’ responses were nonetheless influenced by compatibility between properties of the music and the interpretation of motion information.

One possible explanation why we did not find evidence for fast and slow music motifs conveying motion information has to do with the music stimuli used in the experiments. It is possible that tempo changes in music can convey motion information when the music is well known and the tempo deviations are apparent. For example, playing a well-known tune at both fast and slow exaggerated tempi may convey motion information that simple motifs do not, since most participants would have tempo expectations for such a familiar song and determine how the tempo deviated from their memory of the song’s proper tempo [27]. The fact that simple accelerating and decelerating music motifs were able to convey motion information is supportive of this explanation, as participants were able to gauge the relative change of speed within the course of a single trial. Even though these simple motifs were not well known, participants may have been able to establish an on-the-fly category for each stimulus, and understand the acceleration and deceleration as motion variation.

One issue with the present findings has to do with the distinction between facilitation and interference. Specifically, since we did not use an auditory control, it is difficult to determine whether the response time differences we observed in Experiments 1 and 2 were the result of a facilitation of congruent trials, an interference of incongruent trials, or both. Experiment 3 helps us answer this question, since the music stimuli presumably were not interpreted as containing motion information. In Experiment 3, the response times for all four condition means were slower than the congruent condition means of Experiments 1 and 2. Additionally, three of the four condition means in Experiment 3 were virtually the same as the *incongruent* condition means of Experiments 1 and 2. Thus, while we are not able to conclusively state whether congruent conditions facilitated responses, or incongruent conditions interfered with responses (or both), comparing the means from Experiment 3 to the means from Experiments 1 and 2 provides some evidence that the differences in response times we observed in Experiments 1 and 2 were being driven at least in part by facilitative processes.

The present results also cannot speak to the time course of forming a perceptual representation from comprehension of speech or a signal. Specifically, in our explanation of the results, we hypothesize that listeners engage in mental imagery [4] while listening to musical motifs, which then facilitates object categorization when the implied speed of the visual object and the speed of the musical motif are congruent (“early” time course). It is also possible, however, that listeners are *not* engaged in explicit mental imagery of motion during the presentation of the music, but rather use the motion information contained within the music upon viewing the image (“late” time course). Indeed, the latter possibility would help explain why we did not observe a difference between Experiments 1 and 2. Specifically, if listeners engaged in mental imagery during the music, then the resulting representations could have potentially been stronger (with faster response times) in Experiment 2, since the specific object (e.g., “red car”) was mentioned in the sentence. Overall response times and the magnitude of the interaction, however, were comparable between Experiments 1 and 2 (both *p*>0.5 for overall speed and magnitude of interaction), which potentially supports the “late” time course (in which musical motion is interpreted *after* the presentation of the picture). Future research could shed light on the “early” versus “late” formation of perceptual representations through neural measures, such as functional connectivity (FC) in function magnetic resonance imaging (fMRI). Using FC, one could examine whether auditory areas of the brain (e.g., superior temporal gyrus) are functionally connected to areas implicated in visual mental imagery [28] while listening to musical stimuli, or whether visual areas known for processing motion (e.g., V5) are functionally connected to auditory areas upon viewing the image. The first possibility would support the “early” hypothesis that listeners are forming mental images of motion *before* viewing the image, while the second possibility would support the “late” hypothesis that the auditory motion information influences visual motion perception *after* viewing the image.

The present studies begin to suggest how meaning may be interpreted both in music and in speech for analog acoustic expression. In those situations in which a more continuous description is to be conveyed, speakers can map the variation of specific acoustic properties in speech (e.g., loudness, pitch, timing) onto conceptual properties relevant for communication such as proximity or size, vertical location, or speed or motion of an object in the world [29]. In this respect, analog acoustic expression can function as a separate channel of communication in a conversation much as manual gestures accompanying speech can (see [30]). Moreover, this same type of process appears to be at work as the basis for encoding meaning into music. That observers can make use of analog acoustic information, either in speech or music, demonstrates that this is not some ad hoc strategy but is more likely a basic mechanism by which some classes of intentional signals are interpreted.

The current results can be interpreted in the light of the increasing number of studies that measure music’s ability to convey extramusical information. Koelsch, Kasper, Sammler, Schulze, Gunter, and Friederichi [31] found that music can activate the same brain mechanisms as those involved in the processing of semantic meaning, and that the difference between semantic processing in music and language – as measured though an N400 response – was not statistically different. Furthermore, a related study showed that short and out-of-context musical sounds could also convey meaningful extramusical information, as shown through similar N400 responses as semantic processing in language [32]. In particular, the current results fit into this larger body of literature by suggesting that while certain acoustic features of music may carry semantic information, the mechanism through which this association occurs is not solely driven through perceptual features (i.e. pattern matching). Rather, as the different results in Experiments 2 and 3 suggest, there is a learned component in determining which musical properties will be associated with extramusical information.

The results from the current experiments may also be interpreted in the context of the *shared syntactic integration resource hypothesis* (SSIRH), which states that music and language use the same pool of processing resources for integrating information into syntactic structure [33]. An example of the SSIRH is seen in the studies of Slevc, Rosenberg, & Patel [34], in which the congruency of harmonic progressions influenced the interpretation of garden path sentences. The experiments outlined in this paper expand upon this theory by suggesting that *semantic* properties of both music and language – at least, those represented through temporal change – might similarly share processing resources in the integration of a coherent or complete semantic message. This would explain how listeners were able to use the simultaneously presented music and speech information (Experiment 2) in order to construct a perceptual representation with ease. In support of this idea, previous electrophysiology research has shown that music can prime semantic concepts (e.g. wideness), and linguistic exposure to a contrary concept (e.g. narrowness) elicits an N400 that is comparable to a linguistic prime [31].

Our results are compatible with the idea that language does not simply convey a discrete symbolic-propositional structure, but may be understood in terms of analog perceptual representations [8], [9]. These analog perceptual representations may be influenced by acoustic variation that is separate from the speech signal. In understanding the message of the sentence, the analog variation in accompanying music appears to directly influence how the message is interpreted, creating an expectation about the motion of the described object. Listeners clearly use this expectation, even when it is orthogonal to the task.

Taken together, these results demonstrate that music, like the acoustic properties of speech [7], [12], [35], [36], can convey analog information about objects. Moreover, the way in which music represents motion information appears to be different from the way in which speech represents motion, suggesting that the metaphoric mapping between temporal patterns and motion is nuanced and might dependent on cultural experiences within both musical and linguistic domains.

## Supporting Information

Supporting Information S1 Images from Experiment 1.
(ZIP)

Supporting Information S2 Images from Experiments 2 and 3.
(ZIP)

Supporting Information S3 Sentences from Experiments 2 and 3.
(ZIP)

Supporting Information S4 Music from Experiments 1, 2, and 3.
(ZIP)

## References

[1] Gentner D, Namy LL (2006) Analogical processes in language learning. Curr Dir Psychol Sci 15: 297–301.

[2] Slevc LR (2012) Language and music: sound, structure, and meaning. WIREs Cogn Sci 3: 483–492. 10.1002/wcs.1186 26301531

[3] Meyer LB (1956) Emotion and meaning in music. Chicago: University of Chicago Press. 315 p.

[4] Eitan Z, Granot RY (2006) How music moves: Musical parameters and listeners’ images of motion. Music Percept 25: 221–247.

[5] Eitan Z, Tubul N (2010) Musical parameters and children’s images of motion. Music Sci [Special Issue]: 89–111.

[6] Nygaard LC, Cook AE, Namy LL (2009) Sound to meaning correspondences facilitate word learning. Cognition 112: 181–186. 19447384 10.1016/j.cognition.2009.04.001

[7] Shintel H, Nusbaum HC, Okrent A (2006) Analog acoustic expression in speech communication. J Mem Lang 55: 167–177.

[8] Barsalou LW (1999) Perceptual symbol systems. Behav Brain Sci 22: 577–660. 11301525 10.1017/s0140525x99002149

[9] Zwaan RA, Stanfield RA, Yaxley RH (2002) Language comprehenders mentally represent the shape of objects. Psychol Sci 13: 168–171. 11934002 10.1111/1467-9280.00430

[10] Barsalou LW (2008) Grounded cognition. Annu Rev Psychol 59: 617–645. 17705682 10.1146/annurev.psych.59.103006.093639

[11] Beilock SL, Lyons IM, Mattarella-Micke A, Nusbaum HC, Small SL (2008) Sports experience changes the neural processing of action language. Proc Natl Acad Sci U S A 105: 13269–13272. 18765806 10.1073/pnas.0803424105 PMC2527992

[12] Shintel H, Nusbaum HC (2007) The sound of motion in spoken language: Visual information conveyed by acoustic properties of speech. Cognition 105: 681–690. 17196190 10.1016/j.cognition.2006.11.005

[13] Lakoff G, Johnson M (1980) Metaphors we live by. Chicago: University of Chicago Press. 256 p.

[14] Melara RD, Marks LE (1990) Processes underlying dimensional interactions: Correspondences between linguistic and nonlinguistic dimensions. Mem Cognition 18: 477–495. 10.3758/bf03198481 2233261

[15] Marks LE (1987) On cross-modal similarity: Auditory–visual interactions in speeded discrimination. J Exp Psychol Human 13: 384–394. 10.1037//0096-1523.13.3.384 2958587

[16] Freyd J (1983) The mental representation of movement when static stimuli are viewed. Percept Psychophys 33: 575–581. 6622194 10.3758/bf03202940

[17] Kourtzi Z, Kanwisher N (2000) Implied motion activates extrastriate motion-processing areas. Trends Cogn Sci 4: 295–296. 10904253 10.1016/s1364-6613(00)01512-6

[18] Winawer J, Huk AC, Boroditsky L (2008) A motion aftereffect from still photographs depicting motion. Psychol Sci 19: 276–283. 18315801 10.1111/j.1467-9280.2008.02080.x

[19] Baayen RH, Davidson DJ, Bates DM (2008) Mixed-effects modeling with crossed random effects for subjects and items. J Mem Lang 59: 390–412.

[20] Barr DJ, Levy R, Scheepers C, Tily HJ (2013) Random effects structure for confirmatory hypothesis testing: Keep it maximal. J Mem Lang 68: 255–278. 10.1016/j.jml.2012.11.001 PMC3881361 24403724

[21] Treisman AM, Riley JG (1969) Is selective attention selective perception or selective response? A further test. J Exp Psychol 79: 27–34. 5785632 10.1037/h0026890

[22] Wood NL, Cowan N (1995) The cocktail party phenomenon revisited: Attention and memory in the classic selective listening procedure of Cherry (1953). J Exp Psychol Learn 21: 255–260. 10.1037//0096-3445.124.3.243 7673862

[23] Moray N (1959) Attention in dichotic listening: Affective cues and the influence of instructions. Q J Exp Psychol 11: 56–60.

[24] Cherry EC (1953) Some experiments on the recognition of speech, with one and with two ears. J Acoust Soc Am 25: 975–979.

[25] Shintel H, Nusbaum HC (2008) Moving to the speed of sound: Context modulation of the effect of acoustic properties of speech. Cognitive Sci 32: 1063–1074. 10.1080/03640210801897831 21585443

[26] Goldstein AG (1957) Judgments of visual velocity as a function of length of observation time. J Exp Psychol 54: 457–461. 13491773 10.1037/h0044965

[27] Levitin DJ, Cook PR (1996) Memory for musical tempo: Additional evidence that auditory memory is absolute. Percept Psychophys 58: 927–935. 8768187 10.3758/bf03205494

[28] Bartolomeo P (2002) The relationship between visual perception and visual mental imagery: a reappraisal of the neuropsychological evidence. Cortex 38: 357–78. 12146661 10.1016/s0010-9452(08)70665-8

[29] Parise CV, Pavani F (2011) Evidence of sound symbolism in simple vocalizations. Exp Brain Res 214: 373–380. 21901453 10.1007/s00221-011-2836-3

[30] McNeill D (1992) Hand and mind: What gestures reveal about thought. Chicago: University of Chicago Press. 423 p.

[31] Koelsch S, Kasper E, Sammler D, Schulze K, Gunter TC, et al (2004) Music, language, and meaning: Brain signatures of semantic processing. Nat Neurosci 7: 302–307. 14983184 10.1038/nn1197

[32] Painter JG, Koeslch S (2011) Can out-of-context musical sounds convey meaning? An ERP study on the processing of meaning in music. Psychophysiology 48: 245–255. 10.1111/j.1469-8986.2010.01134.x 20883505

[33] Patel AD (2003) Language, music, syntax, and the brain. Nat Neurosci 6: 674–681. 12830158 10.1038/nn1082

[34] Slevc LR, Rosenberg JC, Patel AD (2009) Making psycholinguistics musical: Self-paced reading time evidence for shared processing of linguistic and musical syntax. Psychon B Rev 16: 374–381. 10.3758/16.2.374 PMC2658747 19293110

[35] Aveyard ME (2012) Some consonants sound curvy: Effects of sound symbolism on object recognition. Mem Cognition 40: 83–92. 10.3758/s13421-011-0139-3 21948332

[36] Nuckolls JB (1999) The case for sound symbolism. Annu Rev Psychol 28: 225–252.

