# Supplementary figures and images for: Conveying Movement in Music and Prosody
Source: PLoS One. 2013 Oct 16;8(10):e76744. doi: 10.1371/journal.pone.0076744 (PMC3797746; doi:10.1371/journal.pone.0076744)

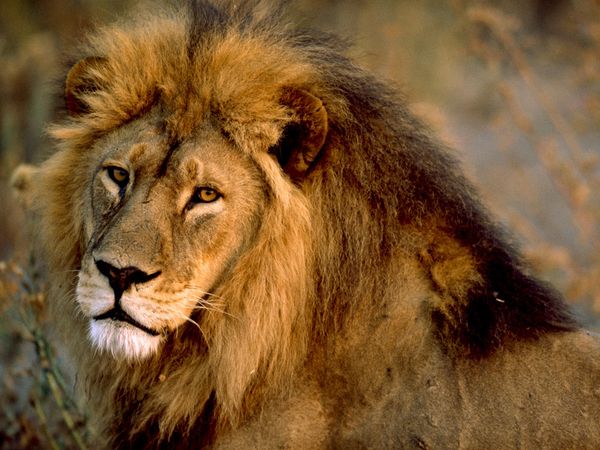

Supplement: Supporting Information S1 — Images from Experiment 1. (ZIP) [file pone.0076744.s001.zip › as10.jpg]

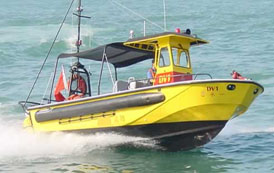

Supplement: Supporting Information S1 — Images from Experiment 1. (ZIP) [file pone.0076744.s001.zip › if1.jpg]

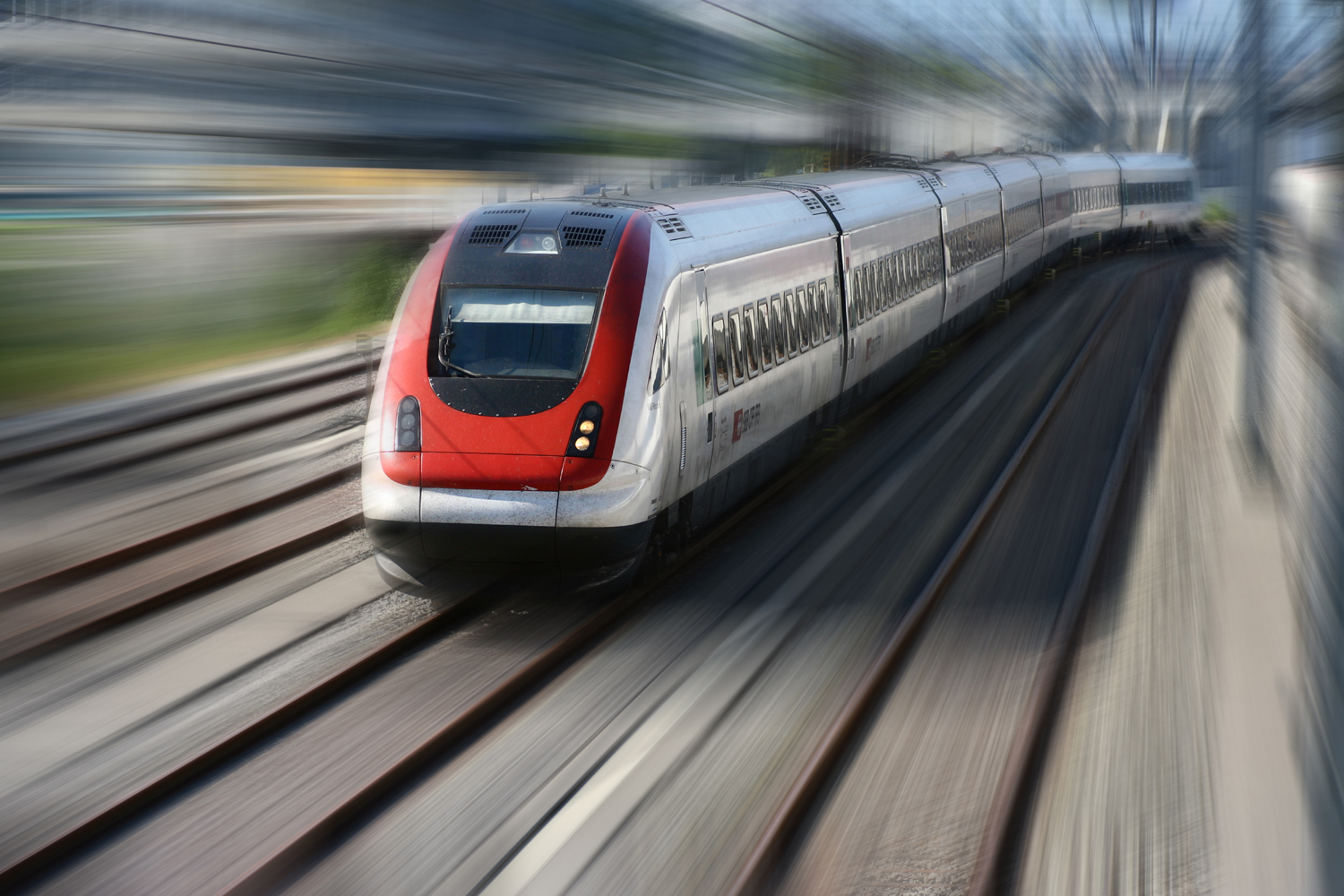

Supplement: Supporting Information S1 — Images from Experiment 1. (ZIP) [file pone.0076744.s001.zip › if3.jpg]

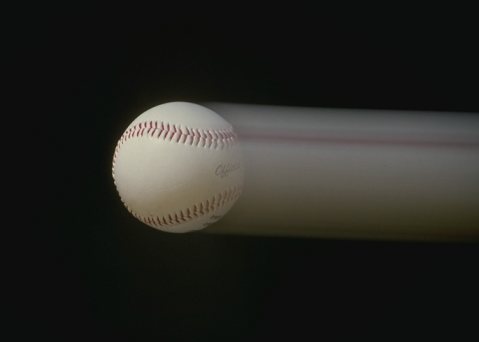

Supplement: Supporting Information S1 — Images from Experiment 1. (ZIP) [file pone.0076744.s001.zip › if4.jpg]

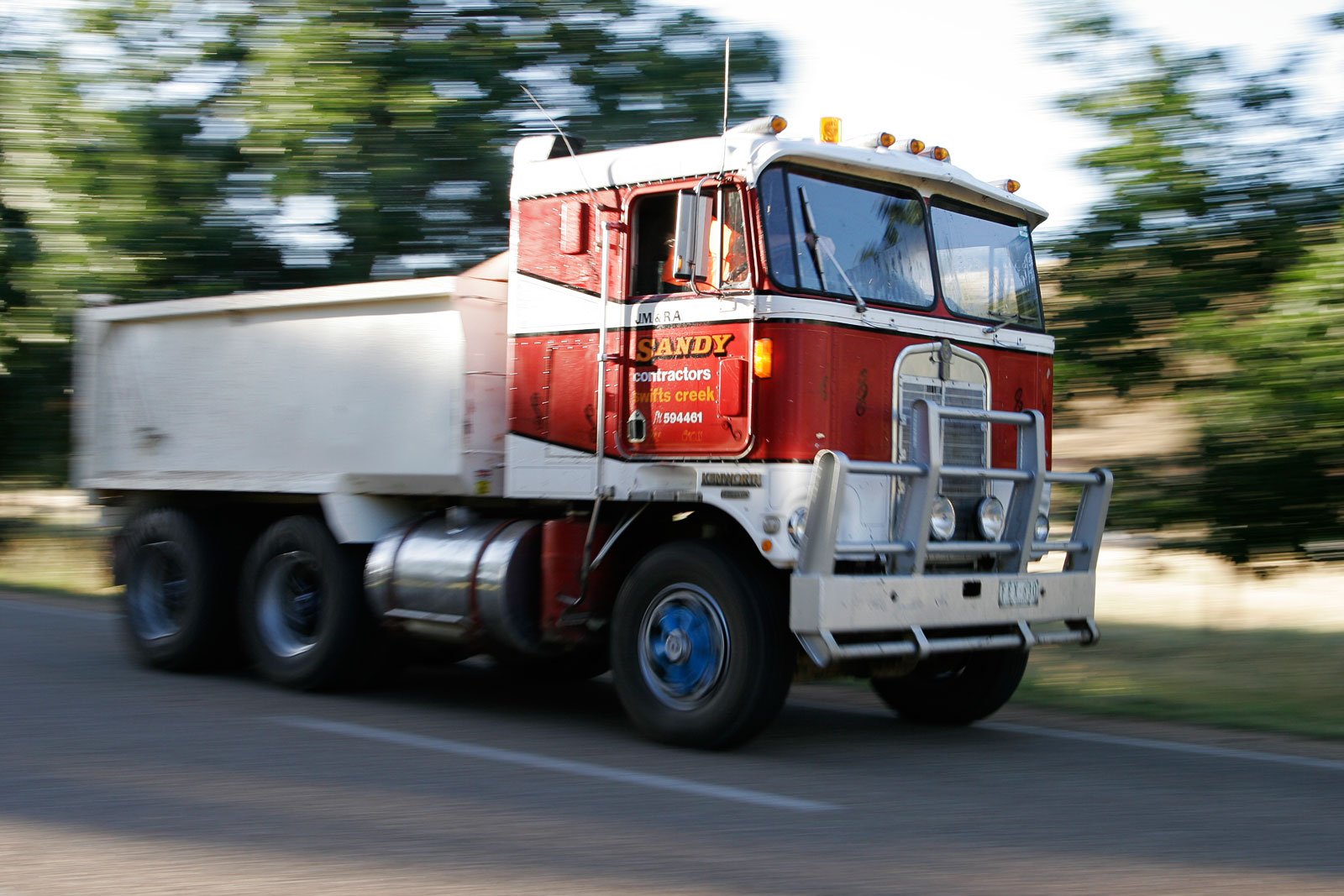

Supplement: Supporting Information S1 — Images from Experiment 1. (ZIP) [file pone.0076744.s001.zip › if5.jpg]

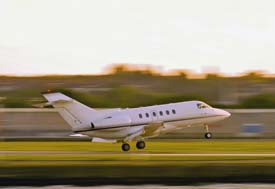

Supplement: Supporting Information S1 — Images from Experiment 1. (ZIP) [file pone.0076744.s001.zip › if6.jpg]

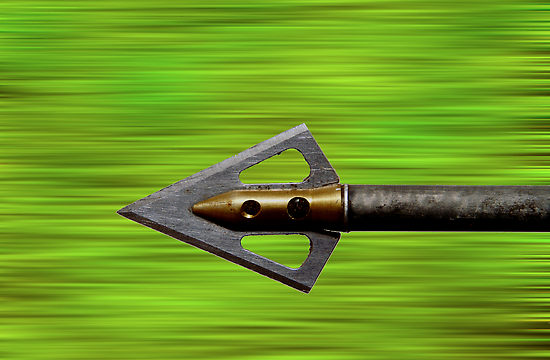

Supplement: Supporting Information S1 — Images from Experiment 1. (ZIP) [file pone.0076744.s001.zip › if7.jpg]

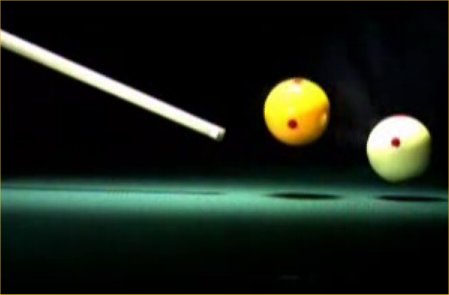

Supplement: Supporting Information S1 — Images from Experiment 1. (ZIP) [file pone.0076744.s001.zip › if8.jpg]

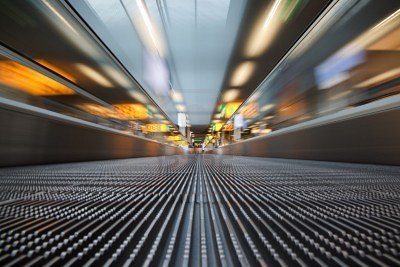

Supplement: Supporting Information S1 — Images from Experiment 1. (ZIP) [file pone.0076744.s001.zip › if9.jpg]

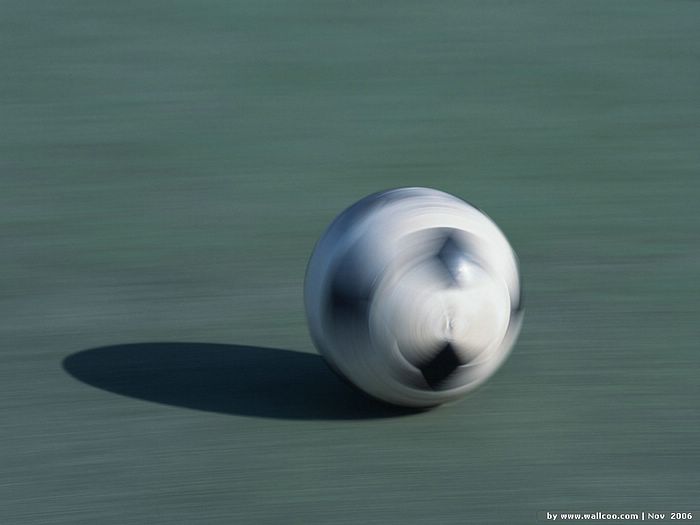

Supplement: Supporting Information S1 — Images from Experiment 1. (ZIP) [file pone.0076744.s001.zip › if10.jpg]

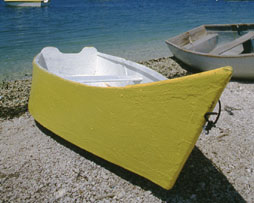

Supplement: Supporting Information S1 — Images from Experiment 1. (ZIP) [file pone.0076744.s001.zip › is1.jpg]

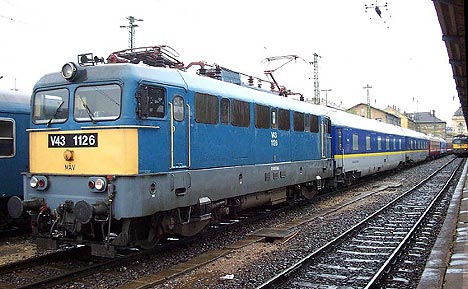

Supplement: Supporting Information S1 — Images from Experiment 1. (ZIP) [file pone.0076744.s001.zip › is3.jpg]

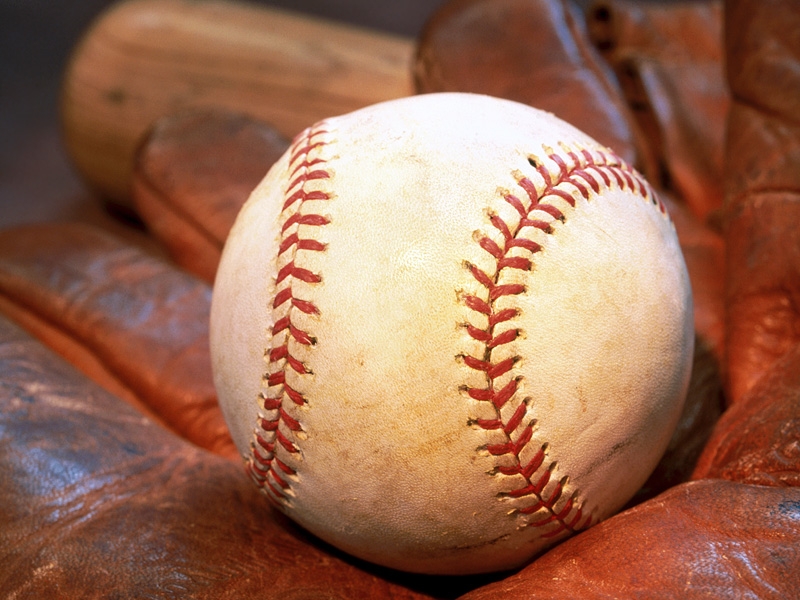

Supplement: Supporting Information S1 — Images from Experiment 1. (ZIP) [file pone.0076744.s001.zip › is4.jpg]

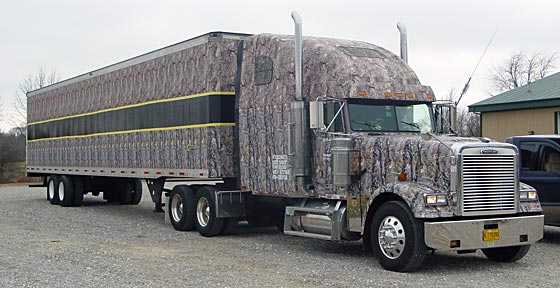

Supplement: Supporting Information S1 — Images from Experiment 1. (ZIP) [file pone.0076744.s001.zip › is5.jpg]

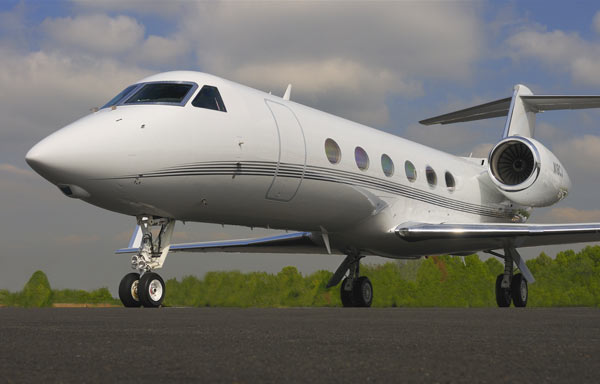

Supplement: Supporting Information S1 — Images from Experiment 1. (ZIP) [file pone.0076744.s001.zip › is6.jpg]

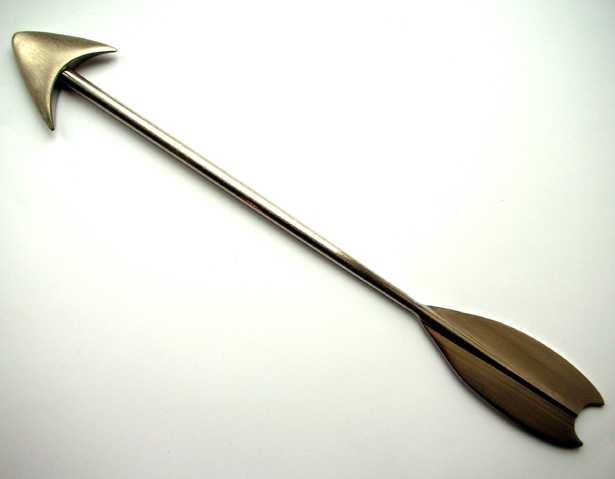

Supplement: Supporting Information S1 — Images from Experiment 1. (ZIP) [file pone.0076744.s001.zip › is7.jpg]

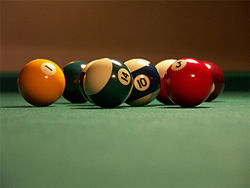

Supplement: Supporting Information S1 — Images from Experiment 1. (ZIP) [file pone.0076744.s001.zip › is8.jpg]

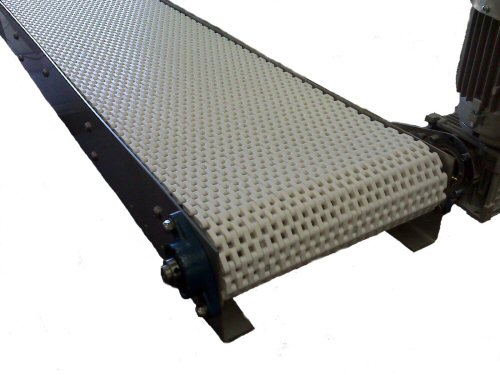

Supplement: Supporting Information S1 — Images from Experiment 1. (ZIP) [file pone.0076744.s001.zip › is9.jpg]

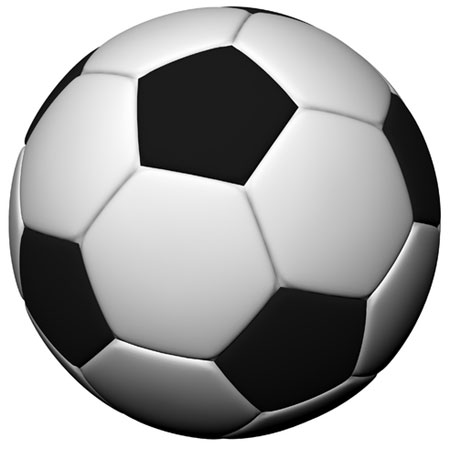

Supplement: Supporting Information S1 — Images from Experiment 1. (ZIP) [file pone.0076744.s001.zip › is10.jpg]

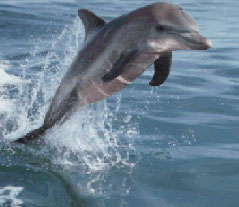

Supplement: Supporting Information S1 — Images from Experiment 1. (ZIP) [file pone.0076744.s001.zip › af6.jpg]

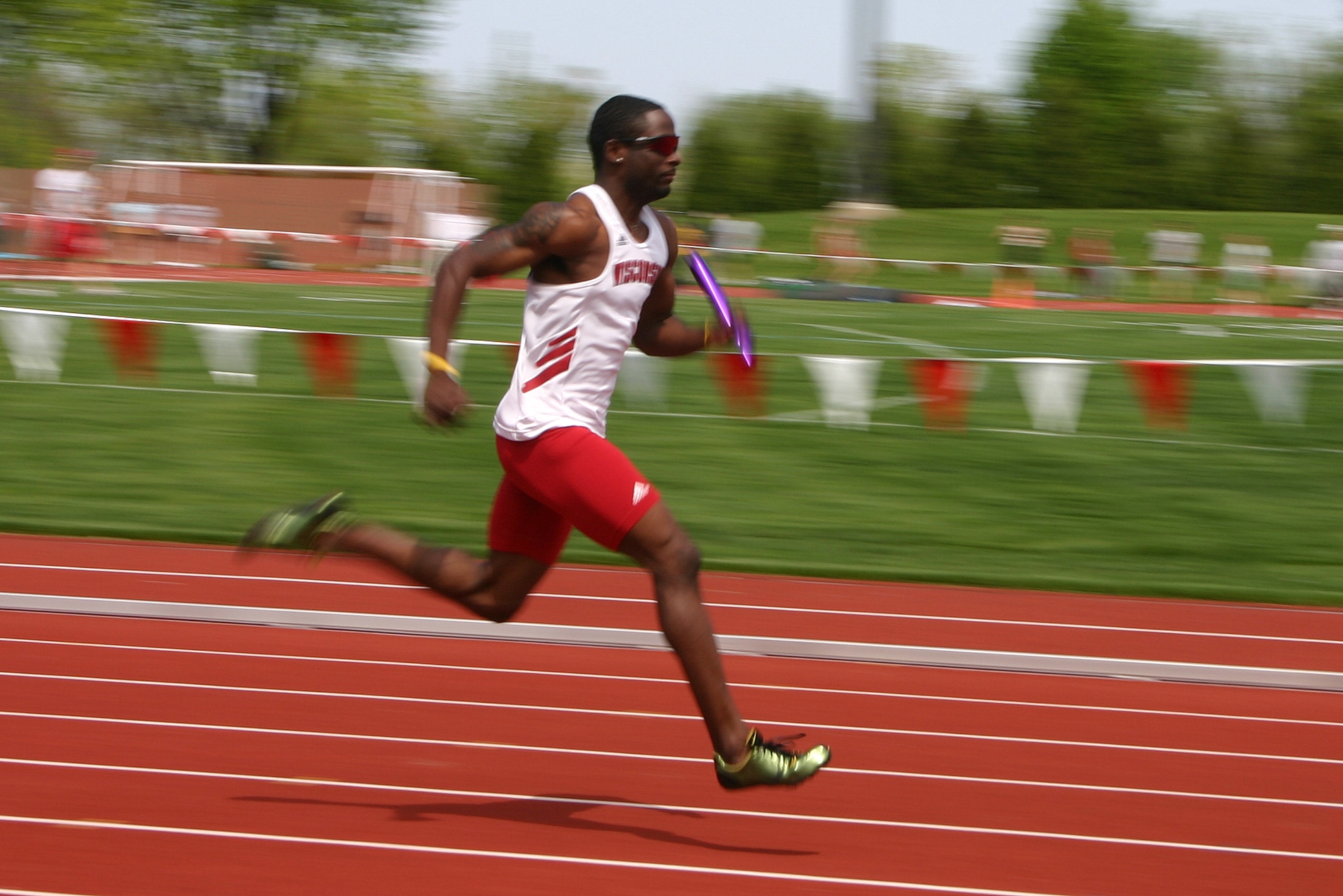

Supplement: Supporting Information S1 — Images from Experiment 1. (ZIP) [file pone.0076744.s001.zip › af8.jpg]

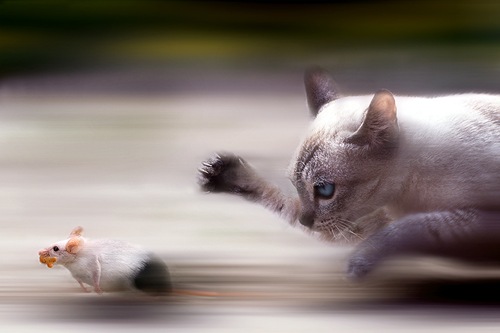

Supplement: Supporting Information S1 — Images from Experiment 1. (ZIP) [file pone.0076744.s001.zip › af9.jpg]

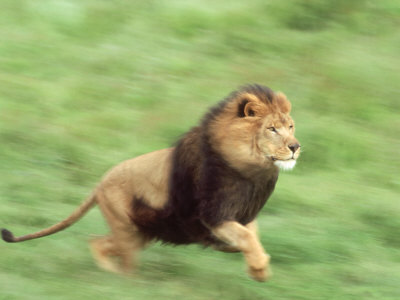

Supplement: Supporting Information S1 — Images from Experiment 1. (ZIP) [file pone.0076744.s001.zip › af10.jpg]

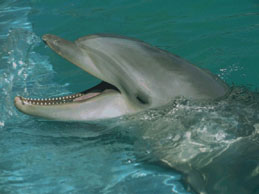

Supplement: Supporting Information S1 — Images from Experiment 1. (ZIP) [file pone.0076744.s001.zip › as6.jpg]

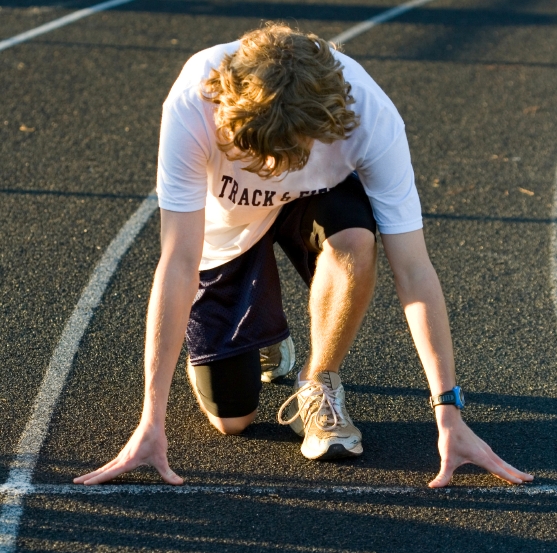

Supplement: Supporting Information S1 — Images from Experiment 1. (ZIP) [file pone.0076744.s001.zip › as8.jpg]

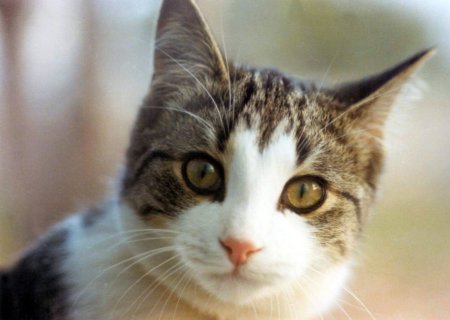

Supplement: Supporting Information S1 — Images from Experiment 1. (ZIP) [file pone.0076744.s001.zip › as9.jpg]

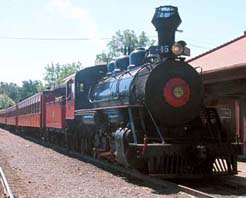

Supplement: Supporting Information S2 — Images from Experiments 2 and 3. (ZIP) [file pone.0076744.s002.zip › train.bmp]

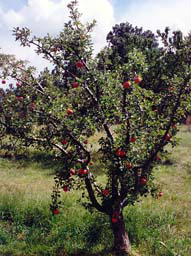

Supplement: Supporting Information S2 — Images from Experiments 2 and 3. (ZIP) [file pone.0076744.s002.zip › appletree.bmp]

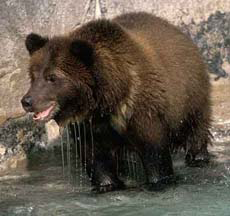

Supplement: Supporting Information S2 — Images from Experiments 2 and 3. (ZIP) [file pone.0076744.s002.zip › bear.bmp]

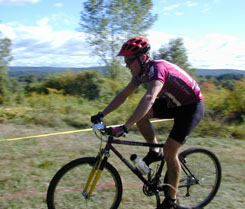

Supplement: Supporting Information S2 — Images from Experiments 2 and 3. (ZIP) [file pone.0076744.s002.zip › bicycleF.bmp]

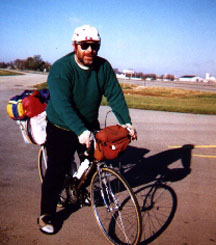

Supplement: Supporting Information S2 — Images from Experiments 2 and 3. (ZIP) [file pone.0076744.s002.zip › bicycleS.bmp]

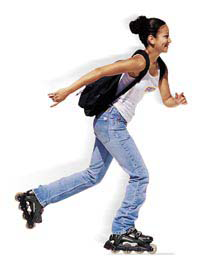

Supplement: Supporting Information S2 — Images from Experiments 2 and 3. (ZIP) [file pone.0076744.s002.zip › bladesF.bmp]

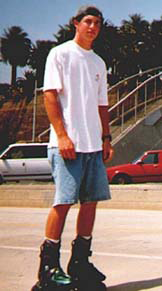

Supplement: Supporting Information S2 — Images from Experiments 2 and 3. (ZIP) [file pone.0076744.s002.zip › bladesS.bmp]

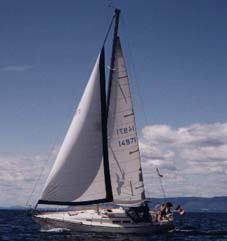

Supplement: Supporting Information S2 — Images from Experiments 2 and 3. (ZIP) [file pone.0076744.s002.zip › boat.bmp]

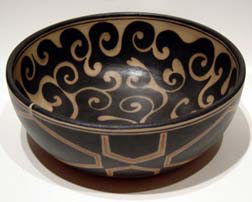

Supplement: Supporting Information S2 — Images from Experiments 2 and 3. (ZIP) [file pone.0076744.s002.zip › bowl.bmp]

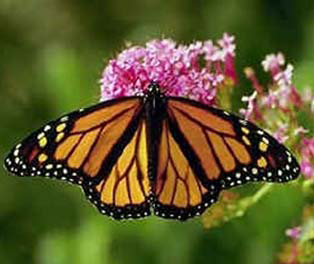

Supplement: Supporting Information S2 — Images from Experiments 2 and 3. (ZIP) [file pone.0076744.s002.zip › butterfly.bmp]

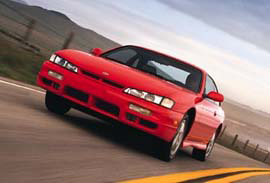

Supplement: Supporting Information S2 — Images from Experiments 2 and 3. (ZIP) [file pone.0076744.s002.zip › carF.bmp]

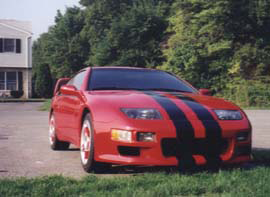

Supplement: Supporting Information S2 — Images from Experiments 2 and 3. (ZIP) [file pone.0076744.s002.zip › carS.bmp]

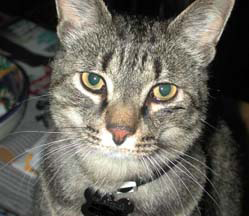

Supplement: Supporting Information S2 — Images from Experiments 2 and 3. (ZIP) [file pone.0076744.s002.zip › cat.bmp]

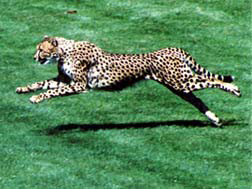

Supplement: Supporting Information S2 — Images from Experiments 2 and 3. (ZIP) [file pone.0076744.s002.zip › cheetahF.bmp]

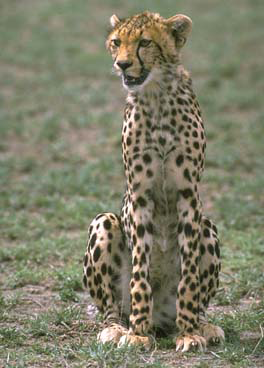

Supplement: Supporting Information S2 — Images from Experiments 2 and 3. (ZIP) [file pone.0076744.s002.zip › cheetahS.bmp]

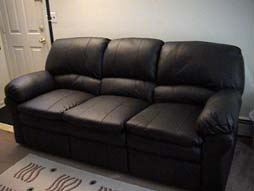

Supplement: Supporting Information S2 — Images from Experiments 2 and 3. (ZIP) [file pone.0076744.s002.zip › couch.bmp]

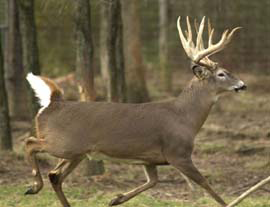

Supplement: Supporting Information S2 — Images from Experiments 2 and 3. (ZIP) [file pone.0076744.s002.zip › deerF.bmp]

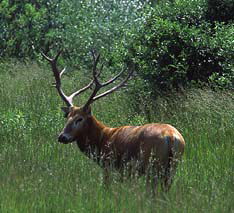

Supplement: Supporting Information S2 — Images from Experiments 2 and 3. (ZIP) [file pone.0076744.s002.zip › deerS.bmp]

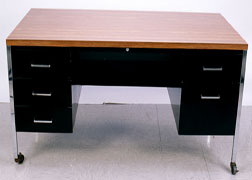

Supplement: Supporting Information S2 — Images from Experiments 2 and 3. (ZIP) [file pone.0076744.s002.zip › desk.bmp]

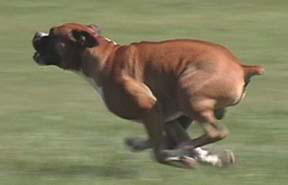

Supplement: Supporting Information S2 — Images from Experiments 2 and 3. (ZIP) [file pone.0076744.s002.zip › dogF.bmp]

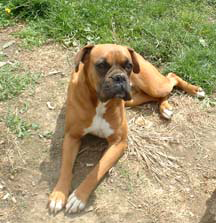

Supplement: Supporting Information S2 — Images from Experiments 2 and 3. (ZIP) [file pone.0076744.s002.zip › dogS.bmp]

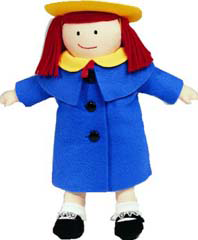

Supplement: Supporting Information S2 — Images from Experiments 2 and 3. (ZIP) [file pone.0076744.s002.zip › doll.bmp]

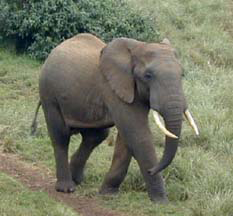

Supplement: Supporting Information S2 — Images from Experiments 2 and 3. (ZIP) [file pone.0076744.s002.zip › elephant.bmp]

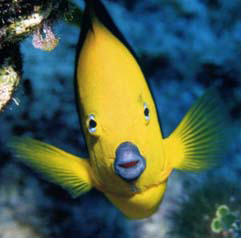

Supplement: Supporting Information S2 — Images from Experiments 2 and 3. (ZIP) [file pone.0076744.s002.zip › fish.bmp]

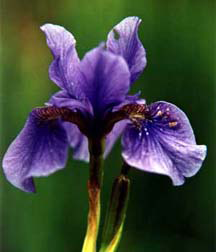

Supplement: Supporting Information S2 — Images from Experiments 2 and 3. (ZIP) [file pone.0076744.s002.zip › flower.bmp]

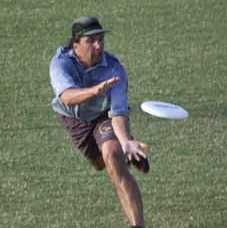

Supplement: Supporting Information S2 — Images from Experiments 2 and 3. (ZIP) [file pone.0076744.s002.zip › frisbeeF.bmp]

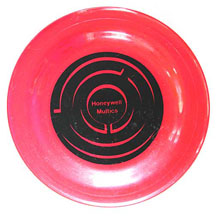

Supplement: Supporting Information S2 — Images from Experiments 2 and 3. (ZIP) [file pone.0076744.s002.zip › frisbeeS.bmp]

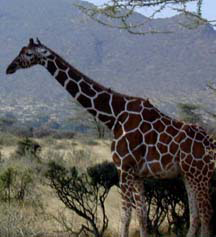

Supplement: Supporting Information S2 — Images from Experiments 2 and 3. (ZIP) [file pone.0076744.s002.zip › giraffe.bmp]

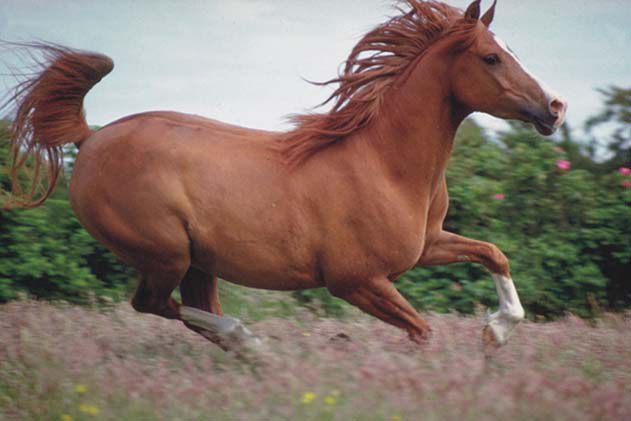

Supplement: Supporting Information S2 — Images from Experiments 2 and 3. (ZIP) [file pone.0076744.s002.zip › horseF.bmp]

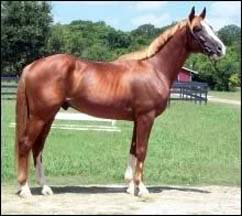

Supplement: Supporting Information S2 — Images from Experiments 2 and 3. (ZIP) [file pone.0076744.s002.zip › horseS.bmp]

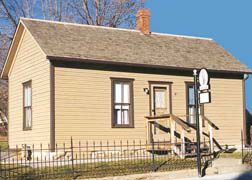

Supplement: Supporting Information S2 — Images from Experiments 2 and 3. (ZIP) [file pone.0076744.s002.zip › House.bmp]

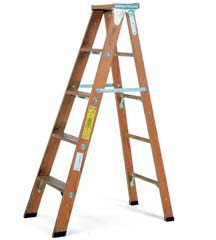

Supplement: Supporting Information S2 — Images from Experiments 2 and 3. (ZIP) [file pone.0076744.s002.zip › ladder.bmp]

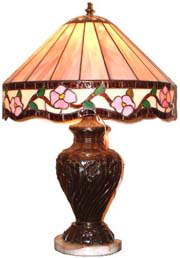

Supplement: Supporting Information S2 — Images from Experiments 2 and 3. (ZIP) [file pone.0076744.s002.zip › lamp.bmp]

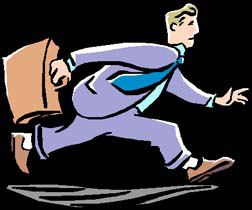

Supplement: Supporting Information S2 — Images from Experiments 2 and 3. (ZIP) [file pone.0076744.s002.zip › manF.bmp]

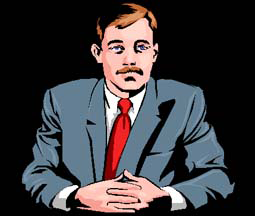

Supplement: Supporting Information S2 — Images from Experiments 2 and 3. (ZIP) [file pone.0076744.s002.zip › manS.bmp]

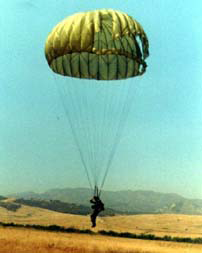

Supplement: Supporting Information S2 — Images from Experiments 2 and 3. (ZIP) [file pone.0076744.s002.zip › parachute.bmp]

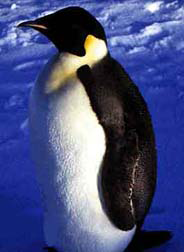

Supplement: Supporting Information S2 — Images from Experiments 2 and 3. (ZIP) [file pone.0076744.s002.zip › penguin.bmp]

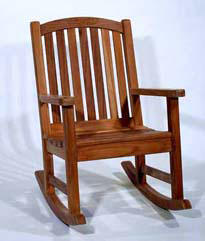

Supplement: Supporting Information S2 — Images from Experiments 2 and 3. (ZIP) [file pone.0076744.s002.zip › rockingchair.bmp]

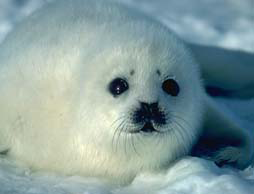

Supplement: Supporting Information S2 — Images from Experiments 2 and 3. (ZIP) [file pone.0076744.s002.zip › seal.bmp]

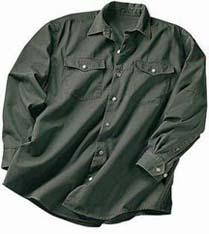

Supplement: Supporting Information S2 — Images from Experiments 2 and 3. (ZIP) [file pone.0076744.s002.zip › shirt.bmp]

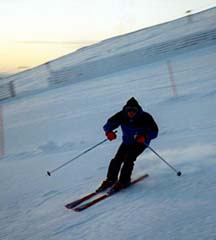

Supplement: Supporting Information S2 — Images from Experiments 2 and 3. (ZIP) [file pone.0076744.s002.zip › skiF.bmp]

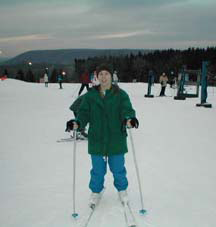

Supplement: Supporting Information S2 — Images from Experiments 2 and 3. (ZIP) [file pone.0076744.s002.zip › skiS.bmp]

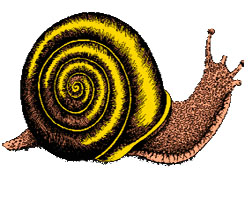

Supplement: Supporting Information S2 — Images from Experiments 2 and 3. (ZIP) [file pone.0076744.s002.zip › snail.bmp]

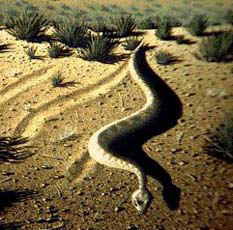

Supplement: Supporting Information S2 — Images from Experiments 2 and 3. (ZIP) [file pone.0076744.s002.zip › SnakeF.bmp]

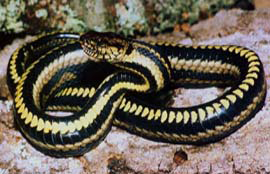

Supplement: Supporting Information S2 — Images from Experiments 2 and 3. (ZIP) [file pone.0076744.s002.zip › snakeS.bmp]

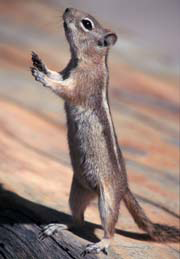

Supplement: Supporting Information S2 — Images from Experiments 2 and 3. (ZIP) [file pone.0076744.s002.zip › squirrel.bmp]

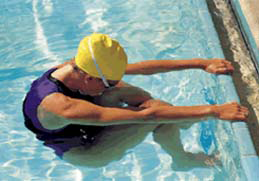

Supplement: Supporting Information S2 — Images from Experiments 2 and 3. (ZIP) [file pone.0076744.s002.zip › swimmerS.bmp]

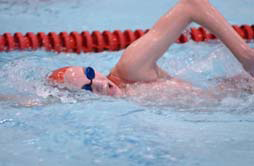

Supplement: Supporting Information S2 — Images from Experiments 2 and 3. (ZIP) [file pone.0076744.s002.zip › swimmingF.bmp]
